# Initial analytical theory of plasma disruption and experimental evidence

**DOI:** 10.1038/s41598-023-36504-7

**Published:** 2023-06-12

**Authors:** Huibin Qiu, Zuozhi Hu, Shengfa Wu, Jiangcun Chen, Chengjie Zhong, Junjie Wu, Xiaobin Li, Donghua Xiao, Chunhui Shi, Junhui Liu, Wenjun Xiong, Tianyi Hu, Qilong Cai, Youlong Yuan

**Affiliations:** 1 Jiangxi Province Key Laboratory of Fusion and Information Control, Department of Physics, Nanchang University, Nanchang, 330031 China; 2 NCU-ASIPP Magnetic Confinement Fusion Joint Lab, Institute of Fusion Energy and Plasma Application, Nanchang University, Nanchang, 330031 China

**Keywords:** Magnetically confined plasmas, Applied physics, Plasma physics, Physics, Statistical physics, thermodynamics and nonlinear dynamics, Nonlinear phenomena, Statistical physics

## Abstract

It is a great physical challenge to achieve controlled nuclear fusion in magnetic confinement tokamak and solve energy shortage problem for decades. In tokamak plasma, large-scale plasma instability called disruption will halt power production of reactor and damage key components. Prediction and prevention of plasma disruption is extremely urgent and important. However, there is no analytical theory can elucidate plasma disruption physical mechanism yet. Here we show an analytical theory of tokamak plasma disruption based on nonextensive geodesic acoustic mode theory, which can give the physical mechanism of disruption. The proposed theory has not only been confirmed by experimental data of disruption on T-10 device, but also can explain many related phenomena around plasma disruption, filling the gap in physical mechanism of tokamak plasma disruption.

## Introduction

Fusion energy provided by magnetic-confinement tokamak reactors holds great promise: sustainability and clean energy^1^. In these reactors^2,3^, avoiding large-scale plasma instabilities called disruptions, which can halt power production and damage critical components, is one of the most pressing challenges^4,5^. Disruptions are especially detrimental to large burning-plasma systems, such as the multibillion-dollar International Thermonuclear Experimental Reactor (ITER) program^6^ currently under construction, which aims to be the first reactor to generate more energy through fusion than injected into the plasma used to heat it^7^. Human beings realize that the prediction and prevention of plasma disruption is extremely urgent and important, and people have indeed conducted a lot of research on the phenomenon of disruption^8,9^, such as the machine learning algorithm that regards the physical mechanism of disruption as a black box to predict disruption^7,10^. However, so far there is no analytical theory that can elucidate the physical mechanism of the plasma disruption phenomenon^7–10^. Here we show an analytical theory of the tokamak plasma disruption, which can give the physical mechanism of the disruption, and present the relevant experimental observational evidence. We assume that the plasma can be described by nonextensive statistical mechanics. On this basis, we establish the nonextensive geodesic acoustic mode theory^11,12^, and through in-depth analysis of this theory, we find that the physical mechanism for the disruption of the tokamak plasma lies in it: when the ion nonextensive parameter is in a specific interval, a strong wave-particle resonance interaction will occur in the plasma, and the wave will continuously absorb energy from the plasma until the amplitude is too large and the plasma disruption occurs. At this time, the ion nonextensive parameter measured by method of ion nonextensive parameter diagnosis^12–14^ is close to $\text {3/5}$, and another accompanying ion nonextensive parameter is close to $\text {1/3}$, which has been confirmed by 59152 shot experimental data on T-10 device. Our results demonstrate that the proposed theory can explain many related phenomena before and after plasma disruption, such as^9,10,15,16^ the conversion of low-frequency waves to high-frequency waves, thermal quench, and current quench. We anticipate that the proposed tokamak plasma disruption theory can become the starting point for a more complex plasma disruption theory. For example, a plasma disruption theory based on the nonextensive gyrokinetic theory, including effects of elongation, triangle deformation, electron and so on, can be developed. In addition, in the tokamak, which is the main device for controlled nuclear fusion, the avoidance of plasma disruption events and the development of more powerful disruption prediction methods will be closely related to such research, such as the utilization of physical quantities like ion nonextensive parameter into the prediction theory^7,10^. These initial results illustrate the potential of nonextensive gyrokinetic theory to accelerate advances in fusion-energy science and, more generally, to understand and predict complex physical systems^12,17–19^.

Tokamak is a device that uses strong magnetic fields to confine high-temperature plasmas, with the aim of creating the conditions for extracting energy from the fusion reactions that take place in the plasma^20^. However, thermal and magnetic energy in the tokamak can drive plasma instabilities leading to disruptions^2^, which is a core science and engineering challenge for nuclear fusion to actually generate electricity^7^. Disruption abruptly disrupts the magnetic confinement of the plasma, terminating the fusion reaction and rapidly depositing plasma energy onto the confining vessel^3,4^. The resulting thermal and electromagnetic force loads can cause irreparable damage to critical device components^7^. The analytical theory of the tokamak plasma disruption is developed on the basis of the nonextensive geodesic acoustic mode theory^12^, and it is an analytical theory that can give the physical mechanism of the plasma disruption phenomenon. It can be used to guide avoiding the generation of plasma disruptions, and at the same time, it provides more positive information for predicting the occurrence of disruptions, and provides new ideas and new solutions for improving the accuracy of prediction. For example, as long as the ion nonextensive parameters are monitored, and by drawing a warning line, the disruptions can be avoided; then combined with deep learning prediction algorithms^7,10^, the occurrence of disruptions can be more accurately predicted. The theories of plasma disruption have been tentatively studied, but there is no analytical theory reliably enough to describe the disruption^7–10^. Currently, there is only some progress in the prediction of disruption from the perspective of machine learning^7,10^.

**Figure 1:**
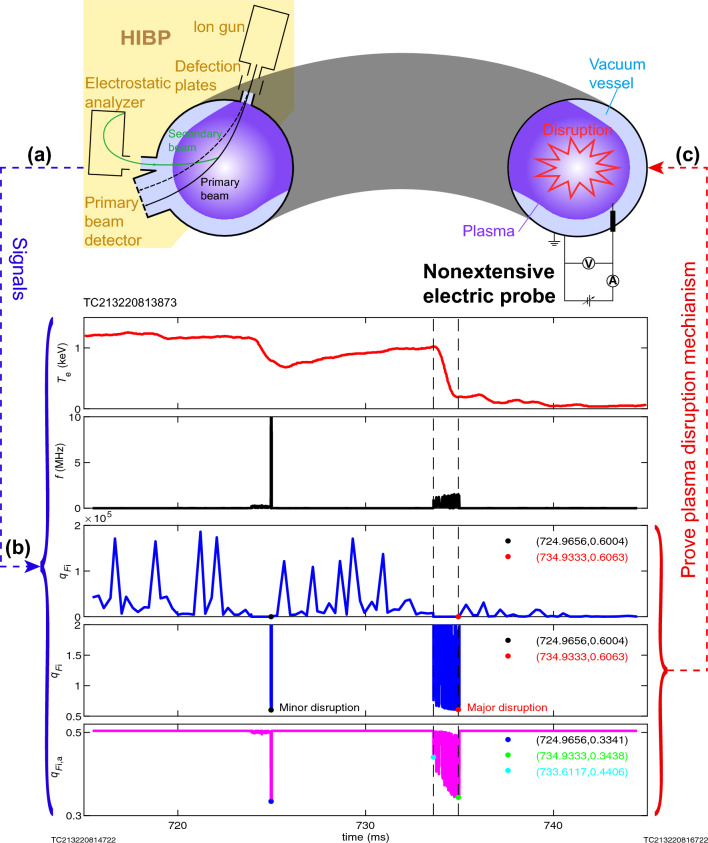
Schematic of test process for tokamak plasma disruption mechanism. (**a**-**c**) The top image shows a circular cross-section tokamak equipped with a plasma electron temperature diagnostic tool—nonextensive electric probe^19,22,23^—and a frequency diagnostic device—heavy ion beam probe (HIBP), and the plasma in the tokamak is undergoing disruption. Diagnostics (**a**) provide sensory data streams (**b**) wherein the plasma electron temperature can be obtained by nonextensive electric probe^19,22,23^, while the frequency signal can be obtained by HIBP, the ion nonextensive parameter (${q_{{F_{\mathrm{{i}}}}}} > {3/5}$) can be given by method of ion nonextensive parameter diagnosis^12–14^, and the accompanying ion nonextensive parameter (${1/ 3}< q_{F_{\textrm{i}},\textrm{a}} < {3/5}$) can be given by the extended ion nonextensive parameter diagnostic method proposed in this work. According to the proposed physical mechanism of tokamak plasma disruption, it is predicted that tokamak plasma disruptions will occur when the (accompanying) ion nonextensive parameters approach (1/3) 3/5. If this is indeed the case, it proves (**c**) that the proposed physical mechanism of tokamak plasma disruption is credible. Panel (**a**) has been modified from a figure of authors’ published paper^12^. ${T_{\mathrm{{e}}}}$, plasma electron temperature; *f*, oscillation frequency of plasma mode; ${q_{{F_{\mathrm{{i}}}}}}$, ion nonextensive parameter; $q_{F_{\textrm{i}},\textrm{a}}$, accompanying ion nonextensive parameter.

However, the development of nonextensive gyrokinetic theory has brought an opportunity for the study of the analytical theory of the disruption: there are increasing evidences that nonextensive statistical mechanics can be considered as the basis for a more appropriate theoretical framework to describe complex systems whose properties cannot be described by Boltzmann–Gibbs statistical mechanics^17,21^. Here, we propose an analytical theory of the plasma disruption based on nonextensive gyrokinetic theory^12^, and give the physical mechanism of the tokamak plasma disruption, which is supported by the experimental data of 59152 shot on T-10 device^15^. We assume that the plasma can be described by nonextensive statistical mechanics, and based on this, we establish the nonextensive geodesic acoustic mode theory^12^, and then, an in-depth analysis of this theory finds that when the ion nonextensive parameter is in a specific interval, a strong wave-particle resonance interaction will occur in the plasma, and the wave will continuously absorb energy from the plasma until the amplitude is too large and the plasma disrupts, that is, a disruption event occurs. The test process of this tokamak plasma disruption mechanism is presented in Fig. 1.

## Results

### Physical mechanism of disruption and prediction

In order to investigate the physical mechanism of tokamak plasma disruption, we make an in-depth research on the theory of nonextensive geodesic acoustic mode^12^ which is based on nonextensive gyrokinetic, and obtain the dispersion relation for the nonextensive (quasi-) geodesic acoustic mode as follows (see “Methods” section):

1 $$\begin{aligned} \omega _{\textrm{r}} = \sqrt{S({{q_F}_{\textrm{i}}},q)}\frac{v_{\textrm{ti}}}{R_0}, \end{aligned}$$

where

2 $$\begin{aligned} {S({{q_F}_{\textrm{i}}},q)}= \frac{1}{2} \left\{ \frac{7}{2(3{{q_F}_{\textrm{i}}}-1)}+\left|-\frac{7}{2(3{{q_F}_{\textrm{i}}}-1)} \right|\sqrt{1+\frac{184}{49q^2}\frac{(3{{q_F}_{\textrm{i}}}-1)}{(5{{q_F}_{\textrm{i}}}-3)}} \right\} , \end{aligned}$$

in which ${q_{{F_{\mathrm{{i}}}}}}$ is the ion nonextensive parameter^12^ that is a real parameter characterizing the nonextensive feature (nonadditivity) of the system^17,24^, and with the physical meaning of the fractal dimension when the Euclidean dimension is one^25,29^.

**Figure 2:**
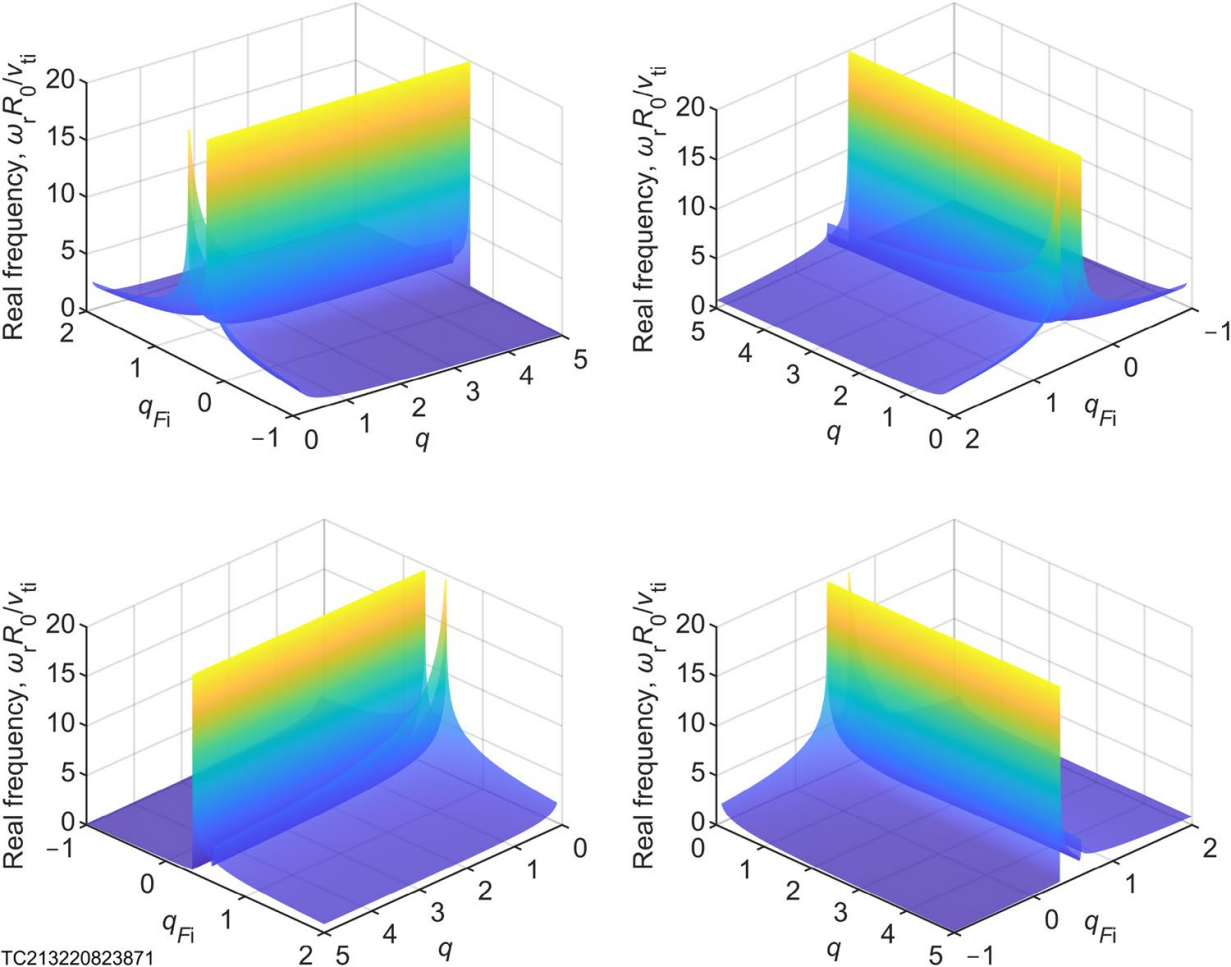
Multi-angle stereograms for physical mechanism of tokamak plasma disruption. There are 2 peaks appearing at ${q_F}_{\textrm{i}} = 3/5$ and ${q_F}_{\textrm{i}} = 1/3$ (see Fig. 3 and Table 1).

The above formula is shown in three-dimensional pictures (Fig. 2). It can be seen that there are two peaks, which are not found in the traditional extensive geodesic acoustic mode theory. Their peaks are infinite. Initially, it seems that there should be new physics in it.

**Figure 3:**
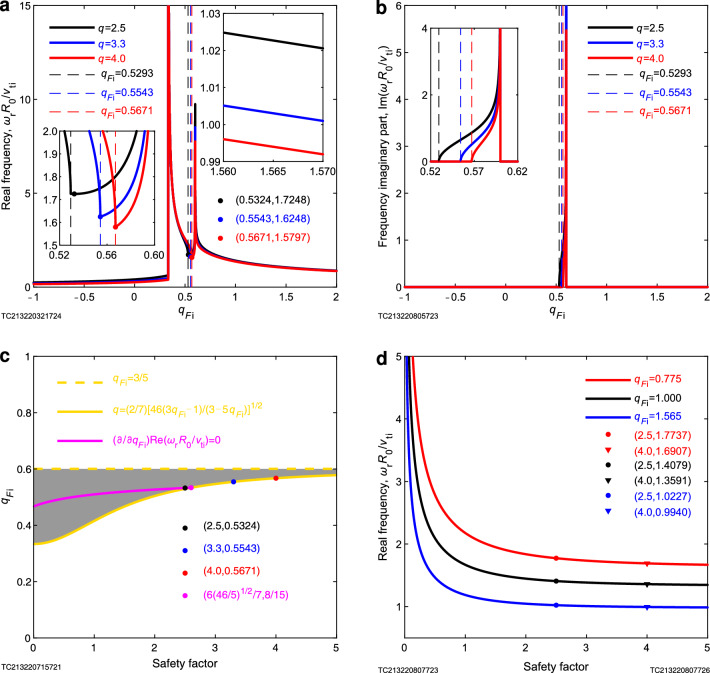
Plane graphics for physical mechanism of tokamak plasma disruption. (**a**-**b**) When the ion nonextensive parameters are close to 3/5 and 1/3, the (quasi-) geodesic acoustic mode frequency tends to infinity, and at $q_{F_{\textrm{i}},\textrm{img}}< {q_F}_{\textrm{i}} \lesssim \mathrm{{3/5}}$, the frequency has a positive imaginary part, which provides a physical mechanism for the plasma disruption. (**c**) Indicate the parameter space where the imaginary frequency occurs (grey area). (**d**) The smaller safety factor is, the disruption is more probably to occur (for details see the information of figure given in “Methods” Section).

In order to clearly illustrate the laws contained in Eq. (1), we use figure (Fig. 3) and table (Table 1) to clearly explain it as follows: it can be seen from Fig. 3a that the frequency of the nonextensive (quasi-) geodesic acoustic mode increases with the decrease of the ion nonextensive parameter, when the safety factor is fixed and the ion nonextensive parameter is more than $\text {3/5}$; as the ion nonextensive parameter is $\text {3/5}$ (see Table 1), the frequency of the quasi-geodesic acoustic mode (quasi-GAM) tends to infinity, which is a mechanism for the conversion of low-frequency waves to high-frequency waves (for example: coupling of (2,1) mode and (1,1) mode^8,9,15,16,26^, and coupling of fast-scale oscillations and (2,1) mode^15^). As can be seen from Fig. 3a–c, when $q_{F_{\textrm{i}},\textrm{img}}< {q_F}_{\textrm{i}} \lesssim \mathrm{{3/5}}$, there is a positive imaginary part of the frequency, and wave-particle resonance occurs, with the wave continuously absorbing energy from the plasma and the amplitude of the wave increasing, at which point disruptions are every probability to occur, and is more probably to occur when the safety factor is small (see Fig. 3d). This suggests that we may have discovered the physical mechanism of tokamak plasma disruption, which is something scientists want to accomplish but haven’t done for decades^7–10^. The above theory is a natural conclusion of the nonextensive geodesic acoustic mode theory^12^ based on nonextensive gyrokinetic, which guarantees the credibility of disruption physical mechanism proposed in this work, while demonstrating the potential of nonextensive gyrokinetic theory. According to the physical mechanism of disruption proposed above, we can give a testable prediction: when the tokamak plasma disruption occurs, the ion nonextensive parameter measured by the method of ion nonextensive parameter diagnosis^12–14^ will be very close to $\text {3/5}$. This will provide us with important methods and ideas for avoiding disruptions and enhancing our ability to the prediction of disruption.

### Evidence for the prediction

To confirm our prediction about the ion nonextensive parameter measured by method of ion nonextensive parameter diagnosis is every close to 3/5 for disruption, we tested it using 59152 shot experimental data on T-10 device.

**Figure 4:**
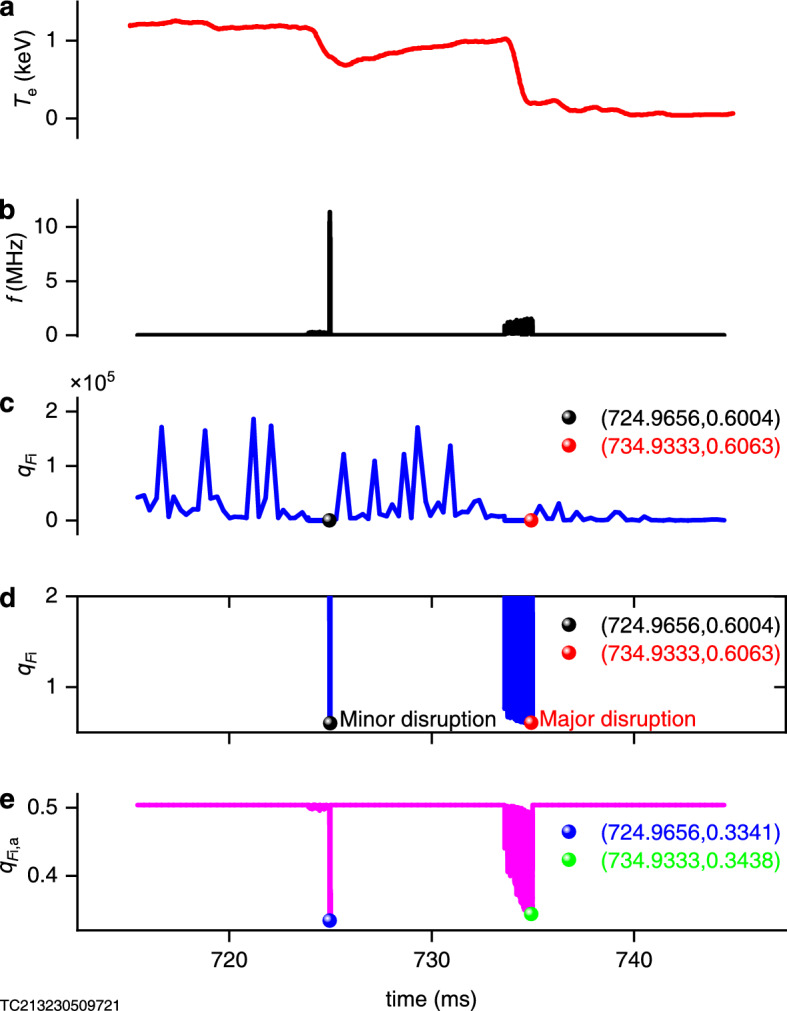
Test of prediction by disruption experimental data on T-10 device. (**a**) Electron temperature experimental data of 59152 shot on T-10 device^15^. (**b**) Mode frequency-time diagram obtained by analyzing the magnetic perturbations experimental data of 59152 shot on T-10 device^15^ (Fig. 5). (**c**) Ion nonextensive parameters measured using the method of ion nonextensive parameter diagnosis^12–14^ in combination with experimental data in (**a**) and (**b**). (**d**) Subplot of (**c**). (**e**) Accompanying ion nonextensive parameters measured after the generation for the method of ion nonextensive parameter diagnostic^12–14^.

**Figure 5:**
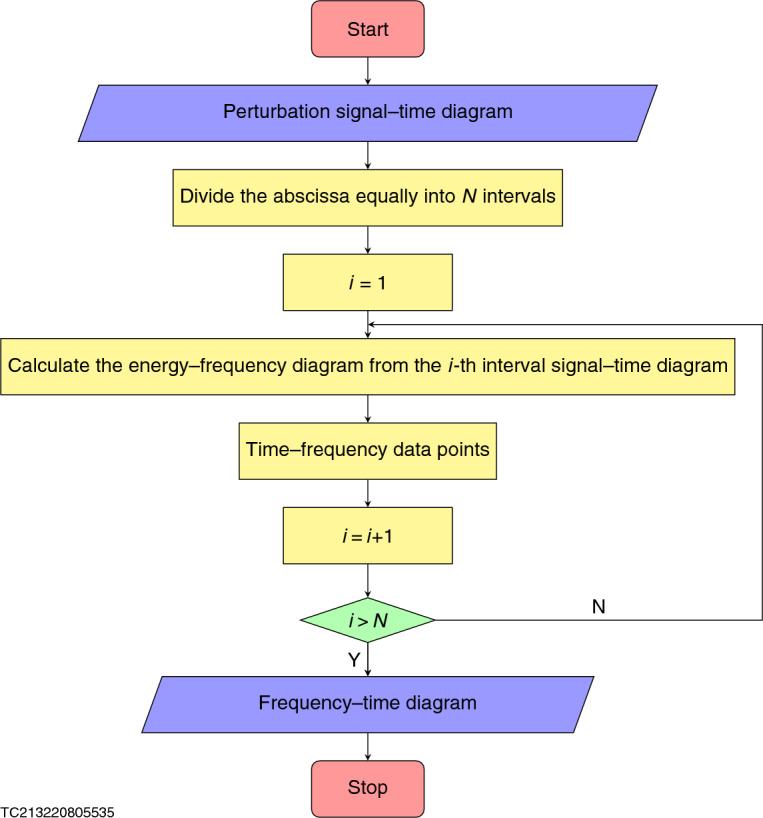
Flow chart of obtaining frequency-time diagram from perturbation signal-time diagram. The frequency-time diagram (Fig. 4b) in this work is obtained from magnetic perturbations-time diagram of Fig. 4 in Ref.^15^ according to the above algorithm.

With the purpose of measuring the plasma ion nonextensive parameters, according to method of ion nonextensive parameter diagnosis^12–14^, it is only necessary to determine the (quasi-) geodesic acoustic mode frequency, plasma electron temperature and safety factor. For 59152 shot on T-10 device, the safety factor is 2^15^. Data on the evolution of plasma electron temperature with time have been provided^15^ (Fig. 4a). Only evolution diagram of the (quasi-) geodesic acoustic mode frequency with time is unknown, but it can be given by analyzing the signal of magnetic perturbations^15^ (Figs. 4b and 5). Using method of ion nonextensive parameter diagnosis^12–14^, Fig. 4c can be given. In order to see more clearly, we construct the subfigure 4d of Fig. 4c. It is obvious from Fig. 4d that when the tokamak plasma disruption, the ion nonextensive parameters are ${q_F}_{\textrm{i}}=0.6004$ and ${q_F}_{\textrm{i}}=0.6063$, they are very close to 3/5, which confirms our above prediction that the ion nonextensive parameters measured by method of ion nonextensive parameter diagnosis^12–14^ will be very close to 3/5 in the case of disruption.

**Table 1:** Data presentation for curve of normalized frequency with ion nonextensive parameter.

*q*$=$2.5	*q*$_{F_\text{i}}$	(–1)$^{+}$	0	(1/3)^−^	(1/3)$^{+}$	0.5293	0.5324	(3/5)^−^	(3/5)$^{+}$	0.775	1	1.565	2	$+\infty$
	$\omega _\text{r}$*R*$_{0}$/*v*$_\text{ti}$	0.2478	0.4089	0.6279	$+\infty$	1.7251$+$0.0004i	1.7248$+$0.2091i	$+\infty +\infty$i	$+\infty$	1.7737	1.4079	1.0227	0.8766	0
*q*$=$3.3	*q*$_{F_\text{i}}$	(–1)$^{+}$	0	(1/3)^−^	(1/3)$^{+}$	0.5543	0.5543	(3/5)^−^	(3/5)$^{+}$	0.775	1	1.565	2	$+\infty$
	$\omega _\text{r}$*R*$_{0}$/*v*$_\text{ti}$	0.1903	0.3128	0.4757	$+\infty$	1.6248$+$0.0021i	1.6248$+$0.0021i	$+\infty +\infty$i	$+\infty$	1.7176	1.3747	1.0030	0.8607	0
*q*$=$4.0	*q*$_{F_\text{i}}$	(–1)$^{+}$	0	(1/3)^−^	(1/3)$^{+}$	0.5671	0.5671	(3/5)^−^	(3/5)$^{+}$	0.775	1	1.565	2	$+\infty$
	$\omega _\text{r}$*R*$_{0}$/*v*$_\text{ti}$	0.1580	0.2591	0.3925	$+\infty$	1.5797$+$0.0043i	1.5797$+$0.0043i	$+\infty +\infty$i	$+\infty$	1.6907	1.3591	0.9940	0.8533	0

In addition, we note that when we make a slight generalization on method of ion nonextensive parameter diagnosis, namely, take into account the function of the $1/3<{q_F}_{\textrm{i}}<3/5$ segment in Eq. (2), then it will give us Fig. 4e. It can be seen that the accompanying ion nonextensive parameters are $q_{F_{\textrm{i}},\textrm{a}}=0.3341$ and $q_{F_{\textrm{i}},\textrm{a}}=0.3438$ when the disruptions occur, and they are also very close to 1/3 which is exactly another peak that is not present in the extensive theory (see Fig. 3a). This is also what we expected. Because at this time, $1/3<q_{F_{\textrm{i}},\textrm{a}}<3/5$, namely, the variation interval is small, thus it has a good identification, so it is reserved in this work.

Figure 4 verifies that the prediction is correct, thus proving that the proposed physical mechanism of disruption is also correct. It is indeed that disruption will occur when the imaginary part appears. Because when the measured $q_{F_{\textrm{i}},\textrm{a}}$ or ${q_F}_{\textrm{i}}$ is close to 1/3 or 3/5, the frequency of the wave is high and a very high positive imaginary part appears for the wave corresponding to the $(q_{F_{\textrm{i}},\textrm{img}},\mathrm {3/5})$ interval; in the moment, a strong wave-particle resonance interaction will occur in the plasma, and the energy of the plasma is rapidly transferred to the wave and the amplitude of the wave grows rapidly; when it reaches the critical value, it will lead to plasma disruption.

### Interpretation of phenomena around disruption

In the absence of a disruption, the ion nonextensive parameter of the tokamak plasma is generally ${q_F}_{\textrm{i}}\gtrsim 1$, for example, the ion nonextensive paremeter is 1.565 for the 36815 shot plasma on T-10 tokamak^12^, also see Fig. 4c and d, while disruption occurring, ${q_F}_{\textrm{i}}$ is close to 3/5. It can be predicted that from the “precursor phase” of a disruption to the “thermal quench”, and then to the “current quench”^8–10,15,16,26,27^, ${q_F}_{\textrm{i}}$ is every probability to develop in a sequence from large to small. So that, in this order of ${q_F}_{\textrm{i}}$, theoretically, there will also be a sequence of “precursor phase”–“thermal quench”–“current quench”. In fact, it is true, for example, when ${q_F}_{\textrm{i}}$ decreases from a large number to 3/5, this is the stage where the frequency of (quasi-) geodesic acoustic mode continues to rise, that is, the stage corresponding to the growth of internal MHD activity precursors (the coupling of (2,1) mode and (1,1) mode^8,9,15,16,26,27^), namely, the “precursor phase”. Immediately after that, as ${q_F}_{\textrm{i}}$ is very close to 3/5, due to the high frequency of the wave, the wave corresponding to the $(q_{F_{\textrm{i}},\textrm{img}},\mathrm {3/5})$ interval appears to have a very high positive imaginary part. At this time, strong wave-particle interactions occur, the energy of the plasma is rapidly transferred to the wave and the temperature of the plasma falls rapidly, which corresponds to the “thermal quench”. After the “thermal quench”, the energy of the plasma is transferred to the wave in large quantities and the amplitude of the wave (such as resistive MHD modes^27^) increases rapidly to a critical value, leading to the disruption of the plasma and it’s the “current quench”^10^.

In conclusion, we propose the physical mechanism for disruption (Figs. 2 and 3), and propose verifiable prediction–at the disruption of plasma in tokamak, the ion nonextensive parameter measured by method of ion nonextensive parameter diagnosis^12–14^ will be very close to 3/5. Then, the prediction is confirmed by the 59152 shot experimental data on T-10 device (Fig. 4), which proves the credibility of the proposed physical mechanism for plasma disruptions in tokamak. At the same time, the proposed theory can reasonably explain the phenomena around the plasma disruption, which makes the proposed theory more credible.

## Discussion and conclusion

Our research result has clarified the physical mechanism of tokamak plasma disruptions, which is the key to solving the problem of disruptions on future fusion devices, such as ITER. Missing a true disruption or calling it too late is costly because its damaging effects cannot be eliminated, while triggering false alarms wastes experimental time and resources^4^. When we already know the physical mechanism of the disruption of the tokamak plasma, first of all, we can monitor the ion nonextensive parameters and delineate a safe area so that it does not enter the area where the disruption occurs; secondly, we can incorporate ion nonextensive parameters into predictive systems (such as machine learning^5,6^) as a useful parameter to improve predictive power.

The theory^12^ on which this work is based is solid, the prediction given is accurate (Figs. 3 and 4), the experimental data^15^ used to test the prediction is reliable, and the explanations of the phenomena^8–10,15,16,26,27^ are reasonable. Thus, this work is trustworthy.

Our work fills the gap of the lack of physical mechanism for plasma disruption.

Our research is the starting point of the theoretical study for the plasma disruption in tokamak based on the nonextensive gyrokinetic theory. The theories of plasma disruption which include effects of plasma elongation, triangle deformation or electron are being solved.

In this work, the analytical theory of plasma disruption is taken as an example to highlight the potential of nonextensive gyrokinetic to complement theory, simulation and experiment in analyzing, predicting and controlling highly complex physical systems. With the great progress made in the research of nonextensive statistical mechanics in various disciplines and fields^12,17,19,21,28^, our findings, as well as some related challenges and insights, have clear significance for the applicability of nonextensive gyrokinetic in fusion science.

## Methods

According to Eq. (14) in Ref.^12^, we can get that the frequency of (quasi-) geodesic acoustic mode reads

3 $$\begin{aligned} \omega _{\textrm{r}} = \frac{v_{\textrm{ti}}}{R_0}\sqrt{S({{q_F}_{\textrm{i}}},q)}, \end{aligned}$$

where

4 $$\begin{aligned} \begin{aligned} {S({{q_F}_{\textrm{i}}},q)}&=\frac{1}{2}\left\{ \frac{7}{2(3{{q_F}_{\textrm{i}}}-1)}+\left|-\frac{7}{2(3{{q_F}_{\textrm{i}}}-1)} \right|\sqrt{1+\frac{4}{q^2}\frac{\frac{23}{2(5{q_F}_{\textrm{i}}-3)(3{q_F}_{\textrm{i}}-1)}}{{\left[ - \frac{7}{2(3{q_F}_{\textrm{i}}-1)} \right] }^2}} \right\} \\&=\frac{1}{2} \left\{ \frac{7}{2(3{{q_F}_{\textrm{i}}}-1)}+\left|-\frac{7}{2(3{{q_F}_{\textrm{i}}}-1)} \right|\sqrt{1+\frac{184}{49q^2}\frac{(3{{q_F}_{\textrm{i}}}-1)}{(5{{q_F}_{\textrm{i}}}-3)}} \right\} , \end{aligned} \end{aligned}$$

in which ${q_F}_{\textrm{i}}>-1$ . When ${q_F}_{\textrm{i}}>1/3$, Eq. (4) becomes

5 $$\begin{aligned} {S({{q_F}_{\textrm{i}}},q)}=\frac{7}{4(3{{q_F}_{\textrm{i}}}-1)} \left\{ 1+\sqrt{1+\frac{4}{q^2}\frac{\frac{23}{2(5{q_F}_{\textrm{i}}-3)(3{q_F}_{\textrm{i}}-1)}}{{\left[ - \frac{7}{2(3{q_F}_{\textrm{i}}-1)} \right] }^2}} \right\} , \end{aligned}$$

which is Eq. (16) in Ref.^12^.

Figure 3a is variation trend diagram of normalized (quasi-) geodesic acoustic mode frequency $$\omega _{\textrm{r}} R_{0}\big /v_{\textrm{ti}}$$ with ion nonextensive parameter $${q_F}_{\textrm{i}}$$ under different safety factors. The abscissa $${q_F}_{\textrm{i}}$$ is the ion nonextensive parameter of plasma with a range of $$\left( { - 1,\mathrm{{ + }}\infty } \right)$$. In this figure, due to the limitation of paper size, only the representative interval of $${q_{{F_{\mathrm{{i}}}}}} \in \left( { - 1,2} \right]$$ is drawn. When $${q_{{F_{\mathrm{{i}}}}}} \rightarrow - 1$$, the nonextensive distribution function is uniform distribution; at $$- 1< {q_{{F_{\mathrm{{i}}}}}} < 1$$, it is kappa distribution; at the extension limit of $${q_{{F_{\mathrm{{i}}}}}} = 1$$, it is Maxwellian distribution, and the results in this case return to the results in the Boltzmann-Gibbs statistical framework^29,30^; when $${q_{{F_{\mathrm{{i}}}}}} > 1$$, it is a truncated distribution which has a cutoff in the tail; it is Dirac delta function at $${q_{{F_{\mathrm{{i}}}}}} \rightarrow + \infty$$. This shows that if nonextensive statistical mechanics is used to describe the plasma, it not only has the advantage of covering the results under the framework of Boltzmann-Gibbs statistical mechanics and proving the correctness of the theory itself in the extensive limit, but also has the advantage of obtaining conclusions that can cover at least four other cases^19^. At safety factor $$q = 2.5$$ and $${q_{{F_{\mathrm{{i}}}}}} = {\left( {{1/3}} \right) ^ - }$$, the real part of the normalized mode frequency is finite, which is 0.6279, and there is no imaginary part. 1/3 is a critical point. When the ion nonextensive parameter is $${q_{{F_{\mathrm{{i}}}}}} < {1/3}$$, it corresponds to low-frequency waves, and when the ion nonextensive parameter is $${q_F}_{\textrm{i}} \gtrsim 1/3$$, it corresponds to high-frequency waves. At $${q_{{F_{\mathrm{{i}}}}}} = {\left( {{1/3}} \right) ^ + }$$, the frequency of the mode is infinite and there is no imaginary part, $${q_{{F_{\mathrm{{i}}}}}} = 0.5293$$ is the place where the imaginary part begins to appear (see also figure $${\textrm{Im}} \left( {{{{\omega _{\mathrm{{r}}}}{R_0}}/{{v_{\mathrm{{ti}}}}}}} \right) - {q_{{F_{\mathrm{{i}}}}}}$$, i.e. Fig. 3b), when $${q_{{F_{\mathrm{{i}}}}}} = 0.5324$$, the real part gets the minimum value of 1.7248. When the safety factor is $$q = 3.3$$ and $${q_{{F_{\mathrm{{i}}}}}} = {\left( {{1 / 3}} \right) ^ - }$$, the real part of the mode is finite, which is 0.4757, and there is no imaginary part. 1/3 is the critical point. When the ion nonextensive parameter is $${q_{{F_{\mathrm{{i}}}}}} < {1 / 3}$$, it corresponds to low-frequency waves, and when the ion nonextensive parameter is $${q_F}_{\textrm{i}} \gtrsim 1/3$$, it corresponds to high-frequency waves, and at $${q_{{F_{\mathrm{{i}}}}}} = {\left( {{1/3}} \right) ^ + }$$, the frequency of the mode is infinite. $${q_{{F_{\mathrm{{i}}}}}} = 0.5543$$ is where the imaginary part begins to appear, and it is also where the real part obtains the minimum value (it has been proved that when the safety factor is $$q \ge \frac{6}{7}\sqrt{\frac{{46}}{5}} \simeq 2.6$$, the place where the imaginary part begins to appear is the place where the real part obtains the minimum value, see Figure $${q_{{F_{\mathrm{{i}}}}}} - q$$, namely, Fig. 3c), and the minimum value is 1.6248. When the safety factor is $$q = 4.0$$ and $${q_{{F_{\mathrm{{i}}}}}} = {\left( {{1/3}} \right) ^ - }$$, the real part of the mode is finite, which is 0.3925, and there is no imaginary part. 1/3 is the critical point. When the ion nonextensive parameter is $${q_{{F_{\mathrm{{i}}}}}} < {1/3}$$, it corresponds to low-frequency waves, and when the ion nonextensive parameter is $${q_F}_{\textrm{i}} \gtrsim 1/3$$, it corresponds to high-frequency waves, and at $${q_{{F_{\mathrm{{i}}}}}} = {\left( {{1/3}} \right) ^ + }$$, the frequency of the mode is infinite. $${q_{{F_{\mathrm{{i}}}}}} = 0.5671$$ is where the imaginary part begins to appear, and it is also where the real part obtains the minimum value, which is 1.5797. At $${q_{{F_{\mathrm{{i}}}}}} = {\left( {{3 / 5}} \right) ^ - }$$, the real part of the frequency of the mode is infinite, and there is an imaginary part, and the imaginary part is also infinite, while at $${q_{{F_{\mathrm{{i}}}}}} = {\left( {{3 / 5}} \right) ^ + }$$, the frequency is infinite and there is no imaginary part. The ordinate is the real part of the (quasi-) geodesic acoustic mode frequency $${{{\omega _{\mathrm{{r}}}}{R_0}} / {{v_{\mathrm{{ti}}}}}}$$, which gives the number of vibrations of the geodesic acoustic mode per second. At $$q = 2.5$$, the value range of $${{{\omega _\mathrm{{r}}}{R_0}}/{{v_{\mathrm{{ti}}}}}}$$ is $$\left( {0, + \infty } \right)$$, and at $${q_{{F_\mathrm{{i}}}}} = {\left( { - 1} \right) ^{\mathrm{{ + }}}}$$, $${{{\omega _\mathrm{{r}}}{R_0}}/{{v_{\mathrm{{ti}}}}}} = 0.2478$$; at $${q_{{F_\mathrm{{i}}}}} = 0$$, $${{{\omega _\mathrm{{r}}}{R_0}}/{{v_{\mathrm{{ti}}}}}} = 0.4089$$; when $${q_{{F_\mathrm{{i}}}}} = {\left( {{1/3}} \right) ^ - }$$, its ordinate is 0.6279; at $${q_{{F_\mathrm{{i}}}}} = {\left( {{1 / 3}} \right) ^ + }$$, the ordinate is $$+ \infty$$. $${q_{{F_\mathrm{{i}}}}} = {1 / 3}$$ is a critical point. When the ion nonextensive parameter is $${q_{{F_\mathrm{{i}}}}} < {1/3}$$, it corresponds to low-frequency waves, and when the ion nonextensive parameter is $${q_F}_{\textrm{i}} \gtrsim 1/3$$, it corresponds to high-frequency waves. At $${q_{{F_\mathrm{{i}}}}} = 0.5293$$, the imaginary part of the ordinate begins to appear. At $${q_{{F_\mathrm{{i}}}}} = 0.5324$$, the real part of the ordinate gets a minimum of 1.7248 and the imaginary part is 0.2091. At $${q_{{F_\mathrm{{i}}}}} = {\left( {{3 / 5}} \right) ^ - }$$, the real part of the corresponding ordinate is +∞, there is an imaginary part, and it is also $$+ \infty$$. At $${q_{{F_\mathrm{{i}}}}} = {\left( {{3/5}} \right) ^ + }$$, the corresponding ordinate is $${{{\omega _\mathrm{{r}}}{R_0}} / {{v_{\mathrm{{ti}}}}}} = + \infty$$. When $${q_{{F_\mathrm{{i}}}}} = 2$$, corresponding ordinate $${{{\omega _\mathrm{{r}}}{R_0}} / {{v_{\mathrm{{ti}}}}}} = 0.8766$$. When $${q_{{F_\mathrm{{i}}}}} = + \infty$$, $${{{\omega _\mathrm{{r}}}{R_0}}/{{v_{\mathrm{{ti}}}}}} = 0$$. At $$q = 3.3$$, the value range of $${\omega _\mathrm{{r}}}{R_0}/{v_{\mathrm{{ti}}}}$$ is $$\left( {0, + \infty } \right)$$, and at $${q_{{F_\mathrm{{i}}}}} = {\left( { - 1} \right) ^ + }$$, $${{{\omega _\mathrm{{r}}}{R_0}} / {{v_{\mathrm{{ti}}}}}} = 0.1903$$. When $${q_{{F_\mathrm{{i}}}}} = 0$$, $${{{\omega _\mathrm{{r}}}{R_0}}/{{v_{\mathrm{{ti}}}}}} = 0.3128$$. At $${q_{{F_\mathrm{{i}}}}} = {\left( {{1/3}} \right) ^ - }$$, its ordinate is 0.4757, and when $${q_{{F_\mathrm{{i}}}}} = {\left( {{1 / 3}} \right) ^ + }$$, the ordinate is $$+ \infty$$. $${q_{{F_\mathrm{{i}}}}} = {1 / 3}$$ is a critical point. When the ion nonextensive parameter is $${q_{{F_\mathrm{{i}}}}} < {1 / 3}$$, it corresponds to low-frequency waves, and when the ion nonextensive parameter is $${q_F}_{\textrm{i}} \gtrsim 1/3$$, it corresponds to high-frequency waves. At $${q_{{F_\mathrm{{i}}}}} = 0.5543$$, the imaginary part of the ordinate begins to appear, the real part of the ordinate gets the minimum value of 1.6248, and the imaginary part is 0.0021. When $${q_{{F_\mathrm{{i}}}}} = {\left( {{3 / 5}} \right) ^ - }$$, the real part of its corresponding ordinate is $$+ \infty$$, there is an imaginary part and it is also $$+ \infty$$. When $${q_{{F_\mathrm{{i}}}}} = 2$$, the corresponding ordinate is $${{{\omega _\mathrm{{r}}}{R_0}} / {{v_{\mathrm{{ti}}}}}} = 0.8607$$. When $${q_{{F_\mathrm{{i}}}}} = + \infty$$, the corresponding ordinate is $${{{\omega _\mathrm{{r}}}{R_0}} / {{v_{\mathrm{{ti}}}}}} = 0$$. When $$q = 4.0$$, the value range of $${{{\omega _\mathrm{{r}}}{R_0}} / {{v_{\mathrm{{ti}}}}}}$$ is $$\left( {0, + \infty } \right)$$. When $${q_{{F_\mathrm{{i}}}}} = {\left( { - 1} \right) ^ + }$$, $${{{\omega _\mathrm{{r}}}{R_0}} / {{v_{\mathrm{{ti}}}}}} = 0.1580$$; when $${q_{{F_\mathrm{{i}}}}} = 0$$, $${{{\omega _\mathrm{{r}}}{R_0}}/{{v_{\mathrm{{ti}}}}}} = 0.2591$$; when $${q_{{F_\mathrm{{i}}}}} = {\left( {{1 / 3}} \right) ^ - }$$, its ordinate is 0.3925; when $${q_{{F_\mathrm{{i}}}}} = {\left( {{1 / 3}} \right) ^ + }$$, the ordinate is $$+\infty$$. $${q_{{F_\mathrm{{i}}}}} = {1 / 3}$$ is a critical point. When the ion nonextensive parameter is $${q_{{F_\mathrm{{i}}}}} < {1 / 3}$$, it corresponds to low-frequency waves, and when the ion nonextensive parameter is $${q_F}_{\textrm{i}} \gtrsim 1/3$$, it corresponds to high-frequency waves. At $${q_{{F_\mathrm{{i}}}}} = 0.5671$$, the imaginary part of the ordinate begins to appear, the real part of the ordinate gets a minimum value of 1.5797, and the imaginary part is 0.0043. When $${q_{{F_\mathrm{{i}}}}} = {\left( {{3 / 5}} \right) ^ - }$$, the real part of the corresponding ordinate is $$+\infty$$, there is an imaginary part, and it is also $$+\infty$$. When $${q_{{F_\mathrm{{i}}}}} = {\left( {{3 / 5}} \right) ^ + }$$, the corresponding ordinate $${{{\omega _\mathrm{{r}}}{R_0}} / {{v_{\mathrm{{ti}}}}}} = + \infty$$. At $${q_{{F_\mathrm{{i}}}}} = 2$$, the corresponding ordinate $${{{\omega _\mathrm{{r}}}{R_0}} / {{v_{\mathrm{{ti}}}}}} = 0.8533$$. At $${q_{{F_\mathrm{{i}}}}} = + \infty$$, $${{{\omega _\mathrm{{r}}}{R_0}}/{{v_{\mathrm{{ti}}}}}} = 0$$. The curve has no integral monotonicity. When $${q_{{F_\mathrm{{i}}}}} \in \left( { - 1,{1 / 3}} 
\right)$$, the ordinate increases with the increase of ion nonextensive parameter. The mathematical reason for this trend is $${{\mathrm{{d}}\left( {{{{\omega _\mathrm{{r}}}{R_0}} / {{v_{\mathrm{{ti}}}}}}} \right) }/{\mathrm{{d}}{q_{{F_\mathrm{{i}}}}}}} > 0$$. When $$q = 2.\mathrm{{5}}$$ and $${q_{{F_\mathrm{{i}}}}} \in \left( {{1 / 3},0.5293} \right)$$, the ordinate decreases with the increase of the ion nonextensive parameter until reaching a smaller value at $${q_{{F_\mathrm{{i}}}}} = 0.5\mathrm{{293}}$$. The mathematical reason for this trend is $${{\mathrm{{d}}\left( {{{{\omega _\mathrm{{r}}}{R_0}} / {{v_{\mathrm{{ti}}}}}}} \right) } / {\mathrm{{d}}{q_{{F_\mathrm{{i}}}}}}} < 0$$. At $${q_{{F_\mathrm{{i}}}}} = 0.5293$$, imaginary parts begin to appear in the ordinate, and at $${q_{{F_\mathrm{{i}}}}} = {\left( {{3 / 5}} \right) ^ - }$$, there are imaginary parts too, until $${q_{{F_\mathrm{{i}}}}} = {\left( {{3 / 5}} \right) ^ + }$$, the imaginary parts disappear (see also figure $${q_{{F_\mathrm{{i}}}}} - q$$, i.e. Fig. 3c). When $${q_{{F_\mathrm{{i}}}}} \in \left( {0.5293,{3 / 5}} \right)$$, $${{{\omega _\mathrm{{r}}}{R_0}} / {{v_{\mathrm{{ti}}}}}}$$ has real and imaginary parts, and the real part decreases first and then increases: it decreases from 1.7251 to 1.7248 in interval $${q_{{F_\mathrm{{i}}}}} \in \left( {0.5293,0.5324} \right)$$, and then increases from 1.7248 to infinity in interval $${q_{{F_\mathrm{{i}}}}} \in \left( {0.5324,{3 / 5}} \right)$$ (see the left-hand subgraph of Fig. 3a); the imaginary part increases monotonically (see Fig. 3b). When $$q = 3.3$$ and $${q_{{F_\mathrm{{i}}}}} \in \left( {{1 / 3},0.5543} \right)$$, the ordinate decreases with the increase of ion nonextensive parameter until it reaches a minimum at $${q_{{F_\mathrm{{i}}}}} = 0.554\mathrm{{3}}$$. The mathematical reason for this trend is $${{\mathrm{{d}}\left( {{{{\omega _\mathrm{{r}}}{R_0}} / {{v_{\mathrm{{ti}}}}}}} \right) }/{\mathrm{{d}}{q_{{F_\mathrm{{i}}}}}}} < 0$$. At $${q_{{F_\mathrm{{i}}}}} = 0.5543$$, ordinate imaginary parts begin to appear, and at $${q_{{F_\mathrm{{i}}}}} = {\left( {{3 / 5}} \right) ^ - }$$, there are imaginary parts, until $${q_{{F_\mathrm{{i}}}}} = {\left( {{3 / 5}} \right) ^ + }$$, the imaginary parts disappear (see Figure $${q_{{F_\mathrm{{i}}}}} - q$$, i.e. Figure 3c). When $${q_{{F_\mathrm{{i}}}}} \in \left( {0.5543,{3 / 5}} \right)$$, $${{{\omega _\mathrm{{r}}}{R_0}} / {{v_{\mathrm{{ti}}}}}}$$ has real and imaginary parts, and both the real part (see the left subgraph of Fig. 3a) and the imaginary part (see Fig. 3b) increase monotonically. When $$q = 4.0$$ and $${q_{{F_\mathrm{{i}}}}} \in \left( {{1 / 3},0.5671} \right)$$, the ordinate decreases with the increase of ion nonextensive parameter until it reaches a minimum at $${q_{{F_\mathrm{{i}}}}} = 0.5671$$. The mathematical reason for this trend is $${{\mathrm{{d}}\left( {{{{\omega _\mathrm{{r}}}{R_0}} / {{v_{\mathrm{{ti}}}}}}} \right) } / {\mathrm{{d}}{q_{{F_\mathrm{{i}}}}}}} < 0$$. At $${q_{{F_\mathrm{{i}}}}} = 0.5671$$, ordinate imaginary parts begin to appear, and at $${q_{{F_\mathrm{{i}}}}} = {\left( {{3 / 5}} \right) ^ - }$$, there are imaginary parts until $${q_{{F_\mathrm{{i}}}}} = {\left( {{3 / 5}} \right) ^ + }$$, the imaginary parts disappear (see Figure $${q_{{F_\mathrm{{i}}}}} - q$$, namely, Fig. 3c). When $${q_{{F_\mathrm{{i}}}}} \in \left( {0.5671,{3 / 5}} \right)$$, $${{{\omega _\mathrm{{r}}}{R_0}} / {{v_{\mathrm{{ti}}}}}}$$ has real and imaginary parts, and both the real part (see the left subgraph of Fig. 3a) and the imaginary part (see Fig. 3b) increase monotonically. When $${q_{{F_\mathrm{{i}}}}} \in \left( {{3 / 5}, + \infty } \right)$$, the ordinate monotonically decreases from positive infinity to zero with the increase of ion nonextensive parameter. The mathematical reason for this change trend is $${{\mathrm{{d}}\left( {{{{\omega _\mathrm{{r}}}{R_0}} / {{v_{\mathrm{{ti}}}}}}} \right) } / {\mathrm{{d}}{q_{{F_\mathrm{{i}}}}}}} < 0$$. Physically, the plasma becomes hotter and hotter from absolute zero in the process of the ion nonextensive parameter decreasing from infinity to $$-1$$. In the interval of $${q_{{F_\mathrm{{i}}}}} \in \left( {0.6, + \infty } \right)$$, when the ion nonextensive parameter decreases from infinity to 0.6, the temperature of the plasma increases gradually from absolute zero, the free energy contained in the plasma increases gradually, and the frequency of the geodesic acoustic mode increases gradually. When near $${q_{{F_\mathrm{{i}}}}} = 0.6$$ (right side of 3/5), the geodesic acoustic mode will be converted to high-frequency waves. When $${q_{{F_\mathrm{{i}}}}} \in \left[ {{q_{{F_\mathrm{{i}}},\mathrm{{img}}}},{3 / 5}} \right)$$ (where $${q_{{F_\mathrm{{i}}},\mathrm{{img}}}}$$ represents the ion nonextensive parameter when the imaginary number begins to appear, which is a function of the safety factor *q*, $${q_{{F_\mathrm{{i}}},\mathrm{{img}}}}\left( {q = 2.5} \right) = 0.5324$$, $${q_{{F_\mathrm{{i}}},\mathrm{{img}}}}\left( {q = 3.3} \right) = 0.5543$$, $${q_{{F_\mathrm{{i}}},\mathrm{{img}}}}\left( {q = 4.0} \right) = 0.5671$$, see Fig. 3 c), the temperature of the plasma rises further. At this time, the imaginary part appears, and the intense energy exchange between the plasma particles and waves occurs. At the same time, the frequency of the plasma wave is at a very high level. At this time, resonance occurs, and the plasma is likely to have a disruption. As the ion nonextensive parameter decreases, the intensity of energy exchange decreases until $${q_{{F_\mathrm{{i}}}}} = {q_{{F_\mathrm{{i}}},\mathrm{{img}}}}$$. When $${q_{{F_\mathrm{{i}}}}} \in \left( {{1 / 3},{q_{{F_\mathrm{{i}}},\mathrm{{img}}}}} \right)$$, the plasma temperature and the energy contained further increase, and the geodesic acoustic mode frequency also increases rapidly. When it is near $${q_{{F_\mathrm{{i}}}}} = {1 / 3}$$ (right side of 1/3), the geodesic acoustic mode will be converted into high-frequency waves. When $${q_{{F_\mathrm{{i}}}}} \in \left( {{{ - 1,1}/ 3}} \right)$$, although the plasma energy increases further with the decrease of ion nonextensive parameter, the geodesic acoustic mode frequency does not increase with the increase, but decreases. The reason is that the farther away from the resonance point, the more the frequency tends to the general state. It can be seen that the geodesic acoustic mode frequency contains fruitful information, which can be used not only for the diagnosis of ion nonextensive parameter^12–14^, but also for the diagnosis of other plasma information^31,32^.

For convenience of viewing, the important data in Fig. 3a are summarized in Table 1.

Figure 3b is variation trend diagram of imaginary part of normalized geodesic acoustic mode frequency with ion nonextensive parameter under different safety factors. When the safety factor is $q = 2.5$, at ${q_{{F_\mathrm{{i}}}}} = 0.5293$, the imaginary part of the mode frequency begins to appear; when the safety factor is $q = 3.3$, at ${q_{{F_\mathrm{{i}}}}} = 0.5543$, the imaginary part of the mode frequency begins to appear; when the safety factor is $q = 4.0$, at ${q_{{F_\mathrm{{i}}}}} = 0.5293$, the imaginary part of the mode frequency begins to appear. Until ${q_{{F_\mathrm{{i}}}}} = {\mathrm{{(}}{3 / 5}\mathrm{{)}}^ - }$, the value of the imaginary part of the mode frequency reaches infinity; at ${q_{{F_\mathrm{{i}}}}} = {\mathrm{{(}}{3 / 5}\mathrm{{)}}^ + }$, the imaginary part of the mode frequency is 0. The ordinate is the imaginary part of the geodesic acoustic model frequency ${\textrm{Im}} ({{{\omega _\mathrm{{r}}}{R_0}}/{{v_{\mathrm{{ti}}}}}})$, which represents the growth rate of the wave amplitude. When the safety factor is $q=2.5$, the value range of ${\textrm{Im}} ({{{\omega _\mathrm{{r}}}{R_0}}/{{v_{\mathrm{{ti}}}}}})$ is $\left[ {0, + \infty } \right)$. At ${q_{{F_\mathrm{{i}}}}} = {\mathrm{{(}} - 1\mathrm{{)}}^ + }$, ${\textrm{Im}} ({{{\omega _\mathrm{{r}}}{R_0}} / {{v_{\mathrm{{ti}}}}}}) = 0$; when $- 1< {q_{{F_\mathrm{{i}}}}} < 0.5293$, the value of ${{{\textrm{Im}} ({\omega _\mathrm{{r}}}{R_0}} / {{v_{\mathrm{{ti}}}})}}$ is equal to 0; at ${q_{{F_\mathrm{{i}}}}} = 0.5293$, ${{{\textrm{Im}} ({\omega _\mathrm{{r}}}{R_0}} / {{v_{\mathrm{{ti}}}})}} = 0.0004$; when $0.5293< {q_{{F_\mathrm{{i}}}}} < {3 / 5}$, the value of ${\textrm{Im}} ({{{\omega _\mathrm{{r}}}{R_0}} / {{v_{\mathrm{{ti}}}}}})$ shows an increasing trend; at ${q_{{F_\mathrm{{i}}}}} = {\left( {{3 / 5}} \right) ^ - }$, its ordinate is $+ \infty$; at ${q_{{F_\mathrm{{i}}}}} = {\left( {{3 / 5}} \right) ^ + }$, the ordinate is 0, and when ${3 / 5}< {q_{{F_\mathrm{{i}}}}} < + \infty$, the value of ${\textrm{Im}} ({{{\omega _\mathrm{{r}}}{R_0}}/{{v_{\mathrm{{ti}}}}}})$ is equal to 0. When the safety factor is $q=3.3$, the value range of ${\textrm{Im}} ({{{\omega _\mathrm{{r}}}{R_0}} / {{v_{\mathrm{{ti}}}}}})$ is $\left[ {0, + \infty } \right)$. At ${q_{{F_\mathrm{{i}}}}} = {\left( { - 1} \right) ^ + }$, ${{{\omega _\mathrm{{r}}}{R_0}} / {{v_{\mathrm{{ti}}}}}} = 0$; when $- 1< {q_{{F_\mathrm{{i}}}}} < 0.5543$, the value of ${\textrm{Im}} ({{{\omega _\mathrm{{r}}}{R_0}} / {{v_{\mathrm{{ti}}}}}})$ is equal to 0; at ${q_F}_{\textrm{i}}=0.5543$, ${\textrm{Im}} ({\omega _\mathrm{{r}}}{R_0}/{v_{\mathrm{{ti}}}}) = 0.0021$; when $0.5543< {q_{{F_\mathrm{{i}}}}} < {3 / 5}$, the value of ${\textrm{Im}} ({\omega _\mathrm{{r}}}{R_0}/{v_{\mathrm{{ti}}}})$ shows an increasing trend; at ${q_{{F_\mathrm{{i}}}}} = {\left( {{3 / 5}} \right) ^ - }$, its ordinate is $+ \infty$; at ${q_{{F_\mathrm{{i}}}}} = {\left( {{3 / 5}} \right) ^ + }$, its ordinate is 0; when ${3 / 5}< {q_{{F_\mathrm{{i}}}}} < + \infty$, the value of ${\textrm{Im}} ({\omega _\mathrm{{r}}}{R_0}/{v_{\mathrm{{ti}}}})$ is equal to 0. When the safety factor is $q=4.0$, the value range of ${\omega _\mathrm{{r}}}{R_0}/{v_{\mathrm{{ti}}}}$ is $\left[ {0, + \infty } \right)$. At ${q_{{F_\mathrm{{i}}}}} = {\left( { - 1} \right) ^ + }$, ${\textrm{Im}} ({\omega _\mathrm{{r}}}{R_0} / {{v_{\mathrm{{ti}}}}}) = 0$; when $- 1< {q_{{F_\mathrm{{i}}}}} < 0.5671$, the value of ${\textrm{Im}} ({\omega _\mathrm{{r}}}{R_0}/{{v_{\mathrm{{ti}}}}})$ is equal to 0; at ${q_{{F_\mathrm{{i}}}}} = 0.5671$, ${\textrm{Im}} ({\omega _\mathrm{{r}}}{R_0} / {{v_{\mathrm{{ti}}}}}) = 0.0043$; when $0.5671< {q_{{F_\mathrm{{i}}}}} < {3 / 5}$, the value of ${\textrm{Im}} ({\omega _\mathrm{{r}}}{R_0} / {{v_{\mathrm{{ti}}}}})$ shows an increasing trend; at ${q_{{F_\mathrm{{i}}}}} = {\left( {{3 / 5}} \right) ^ - }$, its ordinate is $+\infty$; at ${q_{{F_\mathrm{{i}}}}} = {\left( {{3 / 5}} \right) ^ + }$, its ordinate is 0; when ${3 / 5}< {q_{{F_\mathrm{{i}}}}} < + \infty$, the value of ${\textrm{Im}} ({\omega _\mathrm{{r}}}{R_0} / {{v_{\mathrm{{ti}}}}})$ is equal to 0. The curve has no an integral monotonicity. When $q=2.5$ and ${q_{{F_\mathrm{{i}}}}} \in \left( { - 1,0.5293} \right)$, the ordinate is equal to 0 and does not change with the change of ion nonextensive parameter; when ${q_{{F_\mathrm{{i}}}}} \in (0.5\mathrm{{293,}}{3 \big / 5})$, the ordinate increases to infinity with the increase of ion nonextensive parameter, and the mathematical reason for this trend is ${\mathrm{{d}}\left( {{\textrm{Im}} ({\omega _\mathrm{{r}}}{R_0} \big / {{v_{\mathrm{{ti}}}}})} \right) }/ {\mathrm{{d}}{q_{{F_\mathrm{{i}}}}}} > 0$; when ${q_{{F_\mathrm{{i}}}}} \in ({3 / 5}\mathrm{{, + }}\infty )$, the value of the ordinate is always 0. When $q=3.3$ and ${q_{{F_\mathrm{{i}}}}} \in \left( { - 1,0.5543} \right)$, the ordinate is equal to 0 and does not change with the change of ion nonextensive parameter; when ${q_{{F_\mathrm{{i}}}}} \in (0.5543\mathrm{{,}}{3 / 5})$, the ordinate increases to infinity with the increase of ion nonextensive parameter, and the mathematical reason for this trend is ${\mathrm{{d}}\left( {{{{\textrm{Im}} ({\omega _\mathrm{{r}}}{R_0}} / {{v_{\mathrm{{ti}}}}}})} \right) }/ {\mathrm{{d}}{q_{{F_\mathrm{{i}}}}}} > 0$; when ${q_{{F_\mathrm{{i}}}}} \in ({3 / 5}\mathrm{{, + }}\infty )$, the value of the ordinate is always 0. When $q=4.0$ and ${q_{{F_\mathrm{{i}}}}} \in \left( { - 1,0.5671} \right)$, the ordinate is equal to 0 and does not change with the change of ion nonextensive parameter; when ${q_{{F_\mathrm{{i}}}}} \in (0.5671\mathrm{{,}}{3 / 5})$, the ordinate increases to infinity with the increase of ion nonextensive parameter, and the mathematical reason for this trend is ${\mathrm{{d}}\left( {\textrm{Im}} ({\omega _\mathrm{{r}}}{R_0} / {{v_{\mathrm{{ti}}}}}) \right) } / {\mathrm{{d}}{q_{{F_\mathrm{{i}}}}}} > 0$; when ${q_{{F_\mathrm{{i}}}}} \in ({3 / 5}\mathrm{{, + }}\infty )$, the value of the ordinate is always 0. The physical reason is that when the ion nonextensive parameter change from large to small, near ${q_{{F_\mathrm{{i}}}}} = {3 \big / 5}$, the plasma suddenly enters a state of extremely strong wave-particle interaction, which is very likely to cause a plasma disruption. As the ion nonextensive parameter decreases from 3/5, the intensity of wave-particle interaction decreases until ${q_{{F_\mathrm{{i}}}}} = {q_{{F_\mathrm{{i}}},\mathrm{{img}}}}$, and this type wave-particle interaction disappears. In the interval of ${q_{{F_\mathrm{{i}}}}} \in \left( { - 1,{q_{{F_\mathrm{{i}}},\mathrm{{img}}}}} \right) \cup \left( {{3 / 5}, + \infty } \right)$, there is no such type wave-particle interaction.

Figure 3c is the distribution diagram of ion nonextensive parameters when the imaginary part of geodesic acoustic mode frequency appears under the condition of continuous change of safety factor. In the figure, the golden solid curve is point set of $\left\{ \left( {q,{q_{{F_\mathrm{{i}}}}}} \right) \vert q = \frac{2}{7}\sqrt{\frac{{46\left( {3{q_{{F_\mathrm{{i}}}}} - 1} \right) }}{{3 - 5{q_{{F_\mathrm{{i}}}}}}}},\frac{1}{3} \le {q_{{F_\mathrm{{i}}}}} < \frac{3}{5} \right\}$, and the golden dotted line is point set of $\left\{ \left( q,{q_F}_{\textrm{i}}\right) \vert q\ge 0,{q_F}_{\textrm{i}}=\frac{3}{5}\right\}$. There is no intersection between the golden solid line and the golden dotted line. The gray area between the two golden lines is the area where the imaginary part of the geodesic acoustic mode frequency appears. Magenta line is point set of $\left\{ \left( q,{q_F}_{\textrm{i}}\right) \vert \frac{\partial }{\partial {q_F}_{\textrm{i}}}\text{Re} \left( \frac{\omega _{\textrm{r}}R_0}{v_{\textrm{ti}}}\right) =0,0\le q<\frac{6}{7}\sqrt{\frac{46}{5}}\right\}$, where $\frac{\partial }{{\partial {q_{{F_\mathrm{{i}}}}}}}{\rm{{Re}}}\left( {\frac{{{\omega _\mathrm{{r}}}R}}{{{v_{\mathrm{{ti}}}}}}} \right) = \frac{{\left\{ { - 15{q_{{F_\mathrm{{i}}}}}\left[ {7 + 2\sqrt{46} \sqrt{\frac{{3{q_{{F_\mathrm{{i}}}}} - 1}}{{{q^2}\left( {3 - 5{q_{{F_\mathrm{{i}}}}}} \right) }}} } \right] + 7\left[ {9 + 2\sqrt{46} \sqrt{\frac{{3{q_{{F_\mathrm{{i}}}}} - 1}}{{{q^2}\left( {3 - 5{q_{{F_\mathrm{{i}}}}}} \right) }}} } \right] } \right\} }}{{4{{\left[ {\frac{{3{q_{{F_\mathrm{{i}}}}} - 1}}{{{q^2}\left( {3 - 5{q_{{F_\mathrm{{i}}}}}} \right) }}} \right] }^{{1 / 4}}}\left( {5{q_{{F_\mathrm{{i}}}}} - 3} \right) \sqrt{92 + \frac{{7\sqrt{46} }}{{\sqrt{\frac{{3{q_{{F_\mathrm{{i}}}}} - 1}}{{{q^2}\left( {3 - 5{q_{{F_\mathrm{{i}}}}}} \right) }}} }}} }}$ ${{{\left( {\frac{1}{{3{q_{{F_\mathrm{{i}}}}} - 1}}} \right) }^{{3 / 2}}}} {\left( {\frac{{23}}{2}} \right) ^{{1 / 4}}}$. Therefore, it can be known that when $0 \le q < \frac{6}{7}\sqrt{\frac{{46}}{5}}$, the real part of the geodesic acoustic mode frequency obtains a minimum value at the magenta line, while when the safety factor is $q \ge \frac{6}{7}\sqrt{\frac{{46}}{5}} \simeq 2.6$, the place where the imaginary part begins to appear is the place where the real part obtains a minimum value.

Figure 3d is the variation trend diagram of real frequency of (quasi-) geodesic acoustic model with safety factor *q* under different ion nonextensive parameters. When the ion nonextensive parameter ${q_{{F_\mathrm{{i}}}}} = 1.000$, the conclusions return to the results under the Boltzmann-Gibbs statistical framework^12^. ${q_{{F_\mathrm{{i}}}}} = 1.565$ is the ion nonextensive parameter for plasma of 36815 shot on T-10 device^12^. The choice of ${q_{{F_\mathrm{{i}}}}} = 0.775$ is to reflect the situation of ${q_{{F_\mathrm{{i}}}}} < 1$. The abscissa is the safety factor of tokamak device with the value range of $\left[ 0,+ \infty \right)$, and $\left[ 0,5\right]$ is taken in this work, where $q=0$ means that the toroidal magnetic field of the tokamak is zero, the reason why the safety factor greater than 5 is not chosen is because the safety factor of T-10 device is generally between 2.5 and 4. If $q < 2.5$, the disruptions become more frequent and the confinement performance degrades relative to the scaling expression^27^. The ordinate is the real part of the normalized frequency of (quasi-) geodesic acoustic mode ${{{\omega _\mathrm{{r}}}{R_0}} / {{v_{\mathrm{{ti}}}}}}$. In the case of ${q_{{F_\mathrm{{i}}}}} = 0.775$, the value range of ${{{\omega _\mathrm{{r}}}{R_0}} / {{v_{\mathrm{{ti}}}}}}$ is $\left( {\mathrm{{0, + }}\infty } \right)$ (Table 1), and the value range of ${{{\omega _\mathrm{{r}}}{R_0}} / {{v_{\mathrm{{ti}}}}}}$ corresponding to the general range of safety factors on T-10 device is $\left[ {1.6907,1.7737} \right]$. In the case of ${q_{{F_\mathrm{{i}}}}} = 1.000$, the value range of ${{{\omega _\mathrm{{r}}}{R_0}} /{{v_{\mathrm{{ti}}}}}}$ is $\left( {\mathrm{{0, + }}\infty } \right)$ (Table 1), and the value range of ${{{\omega _\mathrm{{r}}}{R_0}} / {{v_{\mathrm{{ti}}}}}}$ corresponding to the general range of safety factors on T-10 device is $\left[ {1.3591,1.4079} \right]$. In the case of ${q_{{F_\mathrm{{i}}}}} = 1.565$, the value range of ${{{\omega _\mathrm{{r}}}{R_0}}/ {{v_{\mathrm{{ti}}}}}}$ is $\left( {\mathrm{{0, + }}\infty } \right)$ (Table 1), and the value range of ${{{\omega _\mathrm{{r}}}{R_0}} / {{v_{\mathrm{{ti}}}}}}$ corresponding to the general range of safety factors on T-10 device is $\left[ {0.9940,1.0227} \right]$. It can be seen from the figure that when the ion nonextensive parameters is constant, the overall curve shows a monotonic decreasing trend. The mathematical reason for this trend is that when ${q_{{F_\mathrm{{i}}}}}$ is fixed, ${{\mathrm{{d}}\left( {{{{\omega _\mathrm{{r}}}{R_0}} / {{v_{\mathrm{{ti}}}}}}} \right) } /{\mathrm{{d}}q}} < 0$. Physically, the (quasi-) geodesic acoustic mode frequency decreases with the increase of safety factor *q* when the ion thermal velocity ${v_{\mathrm{{ti}}}}$ and major radius ${R_0}$ are constant. In other words, the smaller the safety factor, the higher the (quasi-) geodesic acoustic mode frequency, the greater the possibility of disruption.

## Data Availability

All the data generated/analyzed during the study are included in this published article.
