# Publisher Correction: Initial analytical theory of plasma disruption and experimental evidence

**DOI:** 10.1038/s41598-023-37153-6

**Published:** 2023-06-20

**Authors:** Huibin Qiu, Zuozhi Hu, Shengfa Wu, Jiangcun Chen, Chengjie Zhong, Junjie Wu, Xiaobin Li, Donghua Xiao, Chunhui Shi, Junhui Liu, Wenjun Xiong, Tianyi Hu, Qilong Cai, Youlong Yuan

**Affiliations:** 1 Jiangxi Province Key Laboratory of Fusion and Information Control, Department of Physics, Nanchang University, Nanchang, 330031 China; 2 NCU-ASIPP Magnetic Confinement Fusion Joint Lab, Institute of Fusion Energy and Plasma Application, Nanchang University, Nanchang, 330031 China

Correction to: *Scientific Reports* https://doi.org/10.1038/s41598-023-36504-7, published online 12 June 2023

The original PDF version of this Article contained errors in Table 1, where data in column 5 was incorrectly stated as “(1/3)” in three instances, and in column 9 as “(3/5)” in three instances. The correct values for column 5 are “$(1/3{)}^{-}$” and for column 9 “$(3/5{)}^{-}$”, respectively. The correct and incorrect values appear below.

Incorrect:

**Table:** 

$$q=2.5$$	(1/3)	(3/5)
$$q=3.3$$	(1/3)	(3/5)
$$q=4.0$$	(1/3)	(3/5)

Correct:

**Table:** 

$q=2.5$	$(1/3{)}^{-}$	$(3/5{)}^{-}$
$q=3.3$	$(1/3{)}^{-}$	$(3/5{)}^{-}$
$q=4.0$	$(1/3{)}^{-}$	$(3/5{)}^{-}$

These errors have now been corrected in the PDF version of the Article; the HTML version was correct from the time of publication.

